# Application of FreezeTB, a targeted nanopore sequencing assay, for identification of drug resistance and lineages among pulmonary tuberculosis cases in Alaska

**DOI:** 10.1128/spectrum.02335-25

**Published:** 2025-11-17

**Authors:** Bryce Inman, Jeremy Butler, Soren George-Nichol, Ganna Kovalenko, Theresa Savidge, Yvette Vergnetti, Catherine Pongratz, Elizabeth Bee, Andrew R. DiNardo, Alexander Kay, Anna Mandalakas, Eric Bortz, Tara E. Ness

**Affiliations:** 1 Department of Biological Sciences, University of Alaska 164504, Anchorage, Alaska, USA; 2 College of Fisheries and Ocean Sciences, University of Alaska 3291, Fairbanks, Alaska, USA; 3 Wen School of Public Health, University of California 8788, Irvine, California, USA; 4 Alaska TB Program, Division of Public Health, Alaska Department of Health 1247 , Anchorage, Alaska, USA; 5 Global Tuberculosis Program, Baylor College of Medicine 3989, Houston, Texas, USA; 6 Radboud University 6029 , Nijmegen, the Netherlands; 7 Baylor Children's Foundation, Mbabane, Eswatini, Swaziland; 8 Division of Clinical Infectious Diseases, Research Center Borstel, Leibniz Lung Center 28413, Borstel, Germany; 9 German Center for Infection Research (DZIF), Partner Site Hamburg-Lübeck-Borstel-Riems, Borstel, Germany; 10 Department of Microbiology, University of Washington School of Medicine 12353 , Seattle, Washington, USA; Assistance Publique-Hopitaux de Paris Universite Paris Saclay, Clamart, France

**Keywords:** nanopore, sequencing, targeted, tNGS, AMR, MDR-TB, DR-TB, Alaska, tuberculosis, TB, spoligotyping, *Mycobacterium tuberculosis*

## Abstract

Alaska has the highest incidence of tuberculosis (TB) in the United States, with 8% mortality while undergoing TB treatment. With a quarter of TB cases lacking sputum culture to enable drug resistance testing, FreezeTB aimed to develop tools tailored to meet the challenges in Alaska while being translatable to other settings. We designed a rapid and cost-effective laboratory workflow and software to identify drug-resistant mutations in *Mycobacterium tuberculosis* using targeted next-generation sequencing (tNGS). FreezeTB, a Fast, Reliable, Economical Evaluation tool to Zap Endemic Tuberculosis, amplifies 22 gene loci associated with resistance to 16 anti-tuberculosis drugs. *M. tuberculosis* isolates from Alaska (2011–2024) were blinded and underwent analysis with FreezeTB, then compared with phenotypic drug susceptibility testing (pDST) and whole genome sequencing (WGS). Compared with WGS (*n* = 79), FreezeTB provided the same mutations in 96% (*n* = 76/79) of samples with 100% lineage agreement (*n* = 79/79). Compared with pDST using Cohen’s kappa, FreezeTB had almost perfect agreement for rifampin (RIF, 0.90; *n* = 97/98) and ethambutol (EMB, 1.00; *n* = 98/98), strong agreement for isoniazid (INH, 0.80; *n* = 88/98), moderate agreement for ethionamide (ETO, 0.78; *n* = 95/98), weak agreement for streptomycin (STR, 0.56; *n* = 95/98), and no agreement for pyrazinamide (PZA, 0.20; *n* = 91/98). Using a portable nanopore sequencer, each sample cost under $30 for sequencing, which included a flow cell, a barcoding kit, a flow cell wash kit, a polymerase, and primers. FreezeTB provides a portable, 1-day laboratory and bioinformatic workflow for sequencing *M. tuberculosis*. Freeze TB can accurately determine mycobacterial lineage and resistance for RIF, INH, ETH, and ETO at a low cost.

**IMPORTANCE:**

Globally, tuberculosis is the leading infectious cause of mortality with 10.8 million new cases and 1.25 million deaths occurring in 2024. Targeted next-generation sequencing (tNGS) is a rapid, cost-effective method for identifying mutations in the *Mycobacterium tuberculosis* genome associated with drug resistance. FreezeTB was created to provide low-cost, portable sequencing and tNGS analysis for drug resistance. FreezeTB selectively amplifies and sequences 24 targets of the *M. tuberculosis* genome that cover regions the WHO has designated as containing mutations conferring drug resistance, as well as provides species confirmation and rapid lineage determination. Freeze TB is a laboratory workflow and free, open-source (OS) low dependency bioinformatic software that can be downloaded onto a computer with no further need for internet access. Using *M. tuberculosis* isolates from Alaska, FreezeTB provided the same mutations in 96% (*n* = 76/79) of samples with 100% lineage agreement (*n* = 79/79) compared with whole genome sequencing.

## INTRODUCTION

Alaska has the highest incidence of tuberculosis (TB) in the United States, with almost one-third of cases in children under 14 years of age. With 8% mortality while undergoing TB treatment and a quarter of Alaska TB cases without a sputum culture to perform any drug resistance testing (1), TB care in Alaska is in need of improved diagnostic tools, which can be translated to other settings. In 2022–2023, as the COVID-19 pandemic waned, Alaska surged to the highest incident rate of TB in the US, with 10.6–13.1 cases per 100,000 population compared with the US rate of 2.5 cases (2). Of the TB cases over 2 years in Alaska (2022–2023), a disproportionate incidence occurred in Alaska Native/American Indian peoples (AN/AI; OR = 31-fold higher chance of TB disease), and the majority of cases occurred in southwest and northern parts of Alaska.^1^ From 2008–2020, using a whole-genome single-nucleotide polymorphism analysis, it was reported that AN/AI communities in Alaska experienced a 3-fold higher incidence of recurrent TB (rTB) than the United States (US) average (11.8% vs. 3.9%), with 2/3 of cases of rTB likely reinfection as opposed to relapse (3). Although multidrug-resistant TB (MDR-TB) cases have not been as frequent in Alaska compared with the global scale, isoniazid resistance has been identified in 5%–7% of strains isolated in Alaska over the last 3 years (1).

Globally, TB is the leading infectious cause of mortality with 10.8 million new cases and 1.25 million deaths occurring in 2024 (4). Multidrug-resistant TB (MDR-TB) increasingly challenges elimination efforts with an ominous 53% overall case detection gap. Furthermore, it is estimated that only 3%–4% of pediatric MDR-TB cases are diagnosed and treated, with 22% of children dying as a result of their infection (4). Diagnostic tools have struggled to make advancements that can provide rapid, cost-effective diagnosis and determination of drug resistance (5). In Alaska, there is an acute need for rapid TB diagnostics, particularly in more remote communities with high TB prevalence (6).

Targeted next-generation sequencing (tNGS) has been endorsed by the World Health Organization (WHO) as a rapid, cost-effective method for identifying mutations in the *Mycobacterium tuberculosis* genome associated with drug resistance (7, 8) and has been successfully applied to sputum culture isolates (9), direct sputum (10), and stool (11). The WHO evaluated three tNGS products (Deeplex-Myc-TB, AmPORE-TB, TBseq) for a variety of drug resistance determinations (5); however, at present, the costs for sequencing alone can range from $120 to $257 (based on geographical region) (5, 12), and many products require a significant equipment investment for a high complexity sequencing device (ONT GridION ~$58,800 USD [13], Illumina MiSeq i100 ~$49,000 US dollars [14]) that often requires internet access for bioinformatic analysis. The cost, internet necessity, and implementation complexity can make this approach prohibitive for many endemic TB settings, especially without pilot data to demonstrate the benefits of tNGS to clinical diagnosis, retrospective epidemiology, or public health interventions.

To address these issues, we created FreezeTB to provide a laboratory workflow that enables the user to purchase a custom multiplex primer pool at a significantly reduced cost (rather than a kit) and use a low-cost, portable sequencing device for tNGS analysis of drug resistance and mycobacterial lineage (The FreezeTB laboratory protocol is availabe at: https://www.protocols.io/view/freezetb-m-tuberculosis-drug-resistance-screening-hd2eb28bf). For data analysis, we developed a free, open-source (OS) low-dependency bioinformatic software that can be downloaded onto a computer with no further need for internet access. These tools are intended to be used for research purposes, such as retrospective community TB surveillance, allowing the generation of pilot data that can be used to evaluate whether investment in a commercial tNGS option is worthwhile. FreezeTB selectively amplifies and sequences 24 targets of the *M. tuberculosis* genome that cover regions the WHO has designated as containing mutations conferring drug resistance (15), as well as provides species confirmation and rapid lineage determination. In collaboration with the Alaska State Public Health Laboratory (ASPHL), FreezeTB was applied to 100 culture isolates from individuals in Alaska with pulmonary TB, to provide validation of the assay on blinded, newly extracted DNA samples.

## MATERIALS AND METHODS

### *M. tuberculosis* isolates

One hundred *M. tuberculosis* isolates biobanked (stored at −70 degrees Celsius) at the ASPHL from October 2011 to March 2024 were selected for the analysis. Samples were determined exempt under institutional review board (IRB) waiver type 4: Exempt, under Alaska Area IRB (00000636; 12 June 2024), University of Alaska Anchorage IRB (00001046; 15 December 2024), Baylor College of Medicine IRB (H-56320; 30 October 2024); research was approved by the Alaska Native Tribal Health Consortium (25 July 2024) and Southcentral Foundation (15 August 2024). No patient health information was associated with the isolates, and the FreezeTB laboratory team was blinded to the pDST data from the isolates until sequencing and the bioinformatic analysis was completed.

Isolates were chosen by a microbiologist based at ASPHL with a predilection for samples containing drug resistance, as determined by BACTEC 960, MGIT that were collected from 2011 to 2024. Per ASPHL protocols, the samples were grown on BBL Middlebrook 7H9 Broth (or BBL MGIT broth) at 37°C and underwent phenotypic drug susceptibility testing (pDST) for streptomycin (STR), isoniazid (INH), rifampin (RIF), pyrazinamide (PZA), and ethambutol (EMB) from 2011 to mid-2022 and underwent testing for INH, RIF, EMB, PZA, levofloxacin (LFX), and moxifloxacin (MFX) from mid-2022 to 2025. Resistance breakpoints were set at INH 0.1 µg/mL (reflex to 0.4 µg/mL if 0.1 µg/mL is resistant), RIF 1.0 µg/mL, EMB 5.0 µg/mL, PZA 100 µg/mL, LFX 1.0 µg/mL, MFX 0.25 µg/mL, and STR 1.0 µg/mL. Isolates were then heat-inactivated (95°C, 30 min) and underwent viability testing before being transferred to UAA.

### Decontamination and extraction

Isolates underwent decontamination and extraction using the DNeasy Blood and Tissue kit (Qiagen, Hilden, Germany). Decontamination was performed to ensure that the decontamination steps do not interfere with downstream sequencing, allowing the exact protocol to translate to direct sputum respiratory samples. The complete protocol can be found under Material S1. In brief, isolates underwent decontamination using NALC-NaOH-Sodium Citrate (0.5% NALC, 2% NaOH, and 14.5% sodium citrate), with a resuspension in phosphate buffer (0.067 M) and then another resuspension in 1× phosphate-buffered saline (137 mM NaCl, 2.7 mM KCl, 10 mM Na2HPO4, and 1.8 mM KH2PO4). Isolates then underwent DNA extraction per the manufacturer’s instructions, skipping an additional heat-kill step since isolates had been heat-killed before transfer (Material S1). After extraction, DNA was stored at −80°C until further use.

### Primer design

Primers were designed using the Integrated DNA Technologies (IDT, Coralville, Iowa, United States of America) Primer Tool, with the *M. tuberculosis* H37Rv genome as the reference template (NC_000962.3). The WHO catalog was used to define target gene regions of interest and ensure the inclusion of all significant gene mutations associated with drug resistance. Each primer was designed to have an annealing temperature of 60°C, a minimum amplicon length of 700, and a maximum amplicon length of 1,300, with amplicons of roughly equal length providing the best chance of equal coverage. The multiplex contains 32 primer pairs targeting gene mutation regions associated with drug resistance per the WHO (15). Only groups 1 and 2 (Associated with Resistance and Associated with Resistance- Interim) (15) were targeted for inclusion based on the WHO criteria. For genes longer than 1,000 base pairs, two primers were tiled to amplify regions of each gene separately. Genes smaller than 1,000 base pairs were amplified with primers that extended beyond the bounds of the particular gene, ensuring each amplicon was roughly 1,000 base pairs. Each individual primer performance was tested using Mycobacterium bovis bacillus Calmette-Guérin (BCG) (2.6  ×  10^6^ CFU) expressing click beetle red luciferase (CBRLuc) supplied by J. Cirillo (CBRLuc BCG; Center for Airborne Pathogen Research and Tuberculosis Imaging, Bryan, TX, USA) as a template, followed by a 1% gel electrophoresis ensuring minimal off-target amplification and the production of expected-length amplicons. Geneious Prime (GraphPad Software, Boston, MA, USA) was used as a primer validation tool. Species and lineage are determined by targeting the heat shock protein gene (hsp65) and previously designed primers targeting the DRa/DRb repeat regions (16).

### Multiplex design and PCR

The primer multiplex, as designed, was ordered through the Oligo Pools service offered by Integrated DNA Technologies (IDT). The pools are ordered containing each primer at a base concentration of 100 nM, with some primer pairs entered multiple times to provide an optimal concentration for successful amplification of at least 50× depth of coverage for each target region (Table 1). Several polymerases were tested, and the NEBNext Ultra II Q5 Master Mix provided the most consistent effectiveness and cost efficiency. The protocol utilizes 25 µL reaction wells, balancing reagent conservation with generating enough amplicon DNA for sequencing applications (Table 2). Thermocycling conditions were set for an initial denaturation of 98°C for 2 min, then 35 cycles of 98°C for 15 s, 60°C for 15 s, 72°C for 1:20 min, and then a final extension at 72°C for 4 min (Table 3). Testing of pooled primers, polymerases, and cycling conditions was done with CBRLuc BCG. The finalized primer multiplex and protocol were applied to the 100 sputum culture isolates provided by ASPHL. Post-amplification, each sample was diluted with 25 microliters of nuclease-free water for a total volume of 50 µL, followed by a bead clean-up step using AMPure XP beads (Beckman Coulter, Indianapolis, Indiana) in a 1:1 ratio before library preparation, with the final bead cleanup step eluting into 20 μL for downstream sequencing.

**TABLE 1:** Sequences for forward and reverse primers for FreezeTB multiplex*^a^*^,^*^b^*

Primer target	Drug	Forward primer	Reverse primer	Final primer concentration in PCR well (uM)
Rv0678_v2	BDQ/CFZ	ATGAGCAGCGGTATCCA	GTGGTGCGAACAACAAGA	0.1
гроB	RIF	TCGATGTCGTTGTCGTTCTC	GCTCCAGGAAGGGAATCATC	0.3
inhA	INH	TCTGGTTAGCGGAATCATCAC	AACGACAGCAGCAGGAC	0.1
ahpc_v2	INH	GGAATGTCGCAACCAAATG	GTGATTGAGCTCAGGTTCAG	0.1
embB	EMB	TGAAACTGCTGGCGATCAT	GGCGTGAACATCAGGAAGAA	0.1
gyrA	LFX/MFX	TTGACATCGAGCAGGAGATG	CTGGTCCTCAATGTTGGAAATG	0.1
gyrB	LFX/MFX	AGCCGCACCTTTCACTATC	ACATCCAGGAACCGAACATC	0.1
eis_v2	AMK	ACGATTCACGGTCTCCA	GATGATCGACCGGGTTTG	0.1
fabG1_v2	INH	GTTACGCTCGTGGACATAC	TGCGTCCTTGTGTTGTG	0.2
pncA_v2	PZA	CGATGAAGGTGTCGTAGAAG	GGATTTGTCGCTCACTACAT	0.3
rplC_v2	LZD	AGAACGTGTATTGCGTCATC	GTCTTCGTCGAGTGGGTA	0.1
rpsL_v2	STR	GATGAGACGAATCGAGTTTGAG	GGGTTTGACATTGTCGAGAG	0.1
rrl	LZD	AAATCCGTCGCTCACTAATCC	GTTAGCACCAGTTCCCTACAC	0.1
embA_v2	EMB	CAAAGACGACTGGTTTAGGG	ATATCGGGTCAGCCAGTC	0.1
tlyA	CAP	GCGGAGAAGGGTTGAGT	GCAGAACACTGCGATGAG	0.2
fgd1	CFZ	GGCATAGGAGTAGAAAGAACTG	TTACCCGTCTGCGATTCT	0.3
fbiA_v2	DLM/PMD	AGGTACTGTCCTGCGATG	CACCACCTTGCTGGTAAC	0.3
fbiB_v2	DLM/PMD	CGAGCAAAGAGACCGATTG	TCGGGAGGTTGATGGTT	0.1
fbiC_1	DLM/PMD	CGCTACTGTTGCGCTTAT	CATTCGTCGCCAGACAC	0.5
fbiC_2	DLM/PMD	CGCAACCCAAATACGTACA	GGCTCCTGCAACAACTATTA	0.3
ddn_v2	DLM/PMD	CCATCATCGAGCGGATTT	TCTCACCCTTGGTGGTAT	0.3
atpE_v2	BDQ	TTTCGTGTTCGTCTGCTAC	GCGTCCTCGATGACTTTAC	0.2
gid	STR	GCTGGAATGGTTCGATAGTTG	CGTCTGCGATCTTCGGA	0.2
pepQ	BDQ/CFZ	GTTCTTGAAGTCAGCAGTGG	CGTAGCGAGTACCGTCAA	0.1
fbiD	DLM	GCCATCCACACACACTAATC	TCTGTGACGGGACTGTTT	0.1
ethA_1	EMB	CGTCTGACTATGGCCTAAAC	CCTCGACTACGACGCTAA	0.1
ethA_2	EMB	GAGTAGCCCTCGTCGTAG	CTTCCTTGGATGGGAAATAGAA	0.1
rrs_v2	STR	CTAAATACCTTTGGCTCCCTT	GCCCACTACAGACAAGAAC	0.1
katG_1	INH	CTCTTAAGGCTGGCAATCTC	GTGATCACAGCCCGATAAC	0.4
katG_2	INH	GACTGTCTCACAGCTAGTTTC	CATGAGCATTATCCCGTACAC	0.3
Dra/Drb	NA	GGTTTTGGGTCTGACGAC	CGAGAGGGGACGGAAAC	0.1
hsp65	NA	ACCAACGATGGTGTGTCCAT	AGTCGCTGTTCTCGATCT	0.1

^*a*^ To obtain correct concentration in oPool for PCR master mix, primers with a desired final concentration of 0.1 μM are entered into the order sheet once, primers with a desired final concentration of 0.2 μM are entered into the order sheet twice, and primers with a desired final concentration of 0.3 μM are entered into the order sheet three times (and so on).

^*b*^ AMK: Amikacin; BDQ: Bedaquiline; CAP: Capreomycin; CFZ: Clofazimine; DLM: Delamanid; EMB: Ethambutol; ETH: Ethionamide; INH: Isoniazid; LFX: Levofloxacin; LZD: Linezolid; MFX: Moxifloxacin; PMD: Pretomanid; STR: Streptomycin.

**TABLE 2:** Mutations associated with drug resistance identified by FreezeTB tNGS and OS software^*a*^

Drug	Gene	Mutation	Instances
RIF	rpoB	Ser450Leu	2
		Gln432Leu	1
		Gln432Leu	1
		Leu430Arg	1
		p.His445Asp	1
INH	inhA	c.-777C > T	8
	katG	p.Ser315Thr	29
		katG_p.Met1x	1
PZA	pncA	p.His57Asp	1
EMB	embB	p.Met306Val	2
		p.Asp354Ala	1
ETO	inhA	c.-777C > T	8

^*a*^ Please note that mutation inhA confers resistance to both INH and ETO.

**TABLE 3:** Cost of sequencing per sample based on currently listed prices for sequencing supplies and relevant supplier and product codes^*a*^

ITEM	Cost per item	Number of samples	Cost per sample	Supplier	Product code
Flow cell (R10), one flow cell purchase	800	40	20	ONT	FLOMIN114
Barcoding kit (V14, 96 sample plates, three plates)	1100	288	3.82	ONT	SQKRBK114.96
Flow cell wash kit XL (assuming 20 samples per run)	432	960	0.45	ONT	EXPWSH004XL
NEBNext Ultra II Q5 Master Mix	423	250	1.69	NEB	M0543S
Primer mix	125	40	3.13	IDT	Custom
**Total Cost Per Sample**			**$29.09**		

^*a*^ ONT: Oxford Nanopore Technologies; NEB: New England Biolabs; IDT: Integrated DNA Technologies.

### Library preparation and sequencing conditions

Each 20 μL of bead-cleaned amplicons was barcoded using a rapid barcoding kit SQK-RBK114.96 and sequenced with an R10 flow cell (R10.4.1) on a portable MinION Mk1B sequencing device (Oxford Nanopore Technologies [17], Oxford, United Kingdom [UK]). Each barcoding kit was utilized per the manufacturer’s instructions. Although the number of isolates per sequencing run can vary, the maximum number of samples to ensure adequate coverage depth within 2 h of sequencing time is 22 samples. The cutoff Q score was set to nine, and sequencing was done using the super accurate (SUP) basecalling mode, with minimum read length to be basecalled set to 200 using ONT MinKnow (Version: 25.03.9).

### FreezeTB software

FreezeTB software was created to be a free, OS software with a graphical user interface (GUI) that can be downloaded and then used without an internet connection to ensure the utmost usability. More information regarding the software development can be found in Material S2.

**Fig 1:**
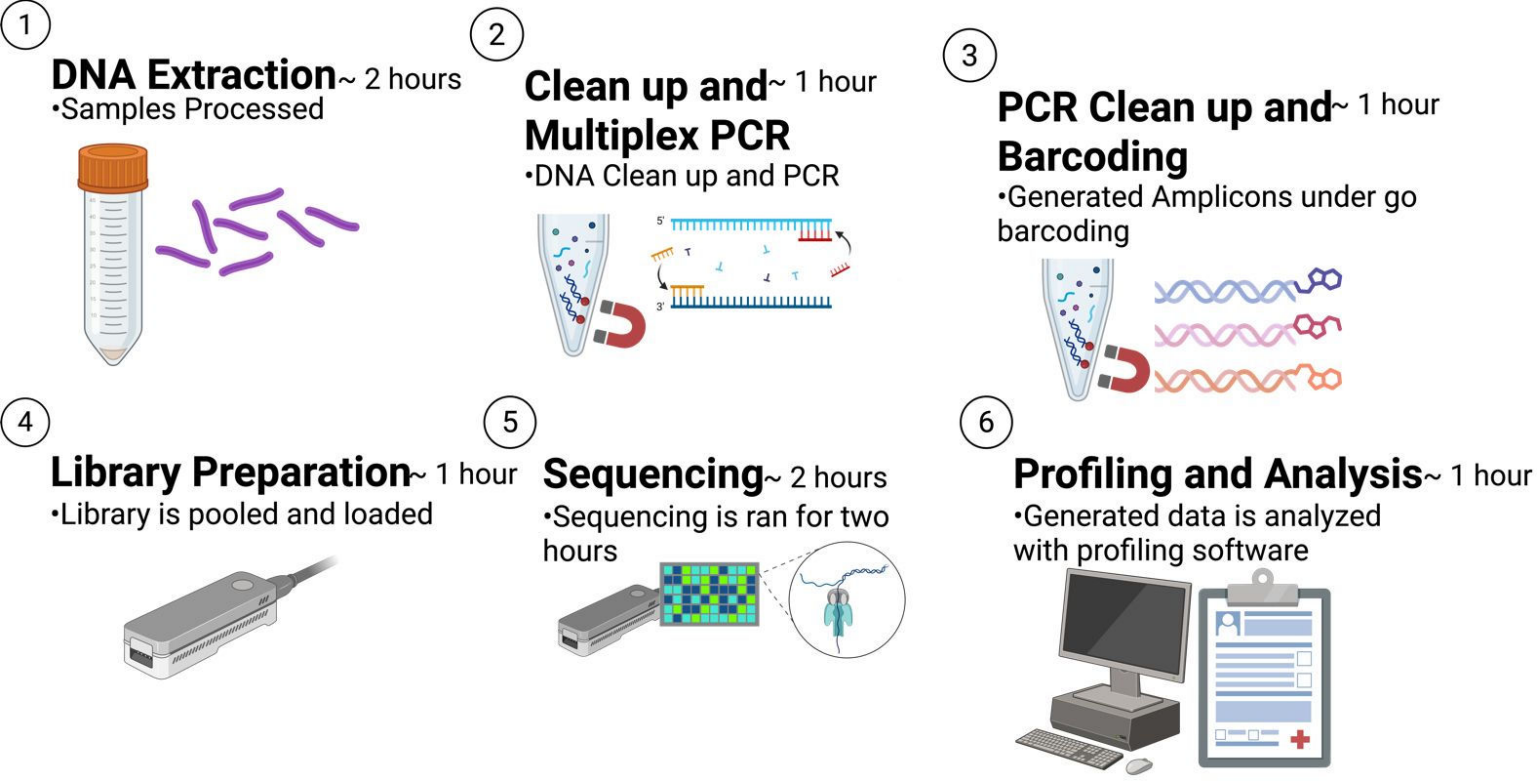
Overall workflow of FreezeTB with relative time spent on each step of the workflow. Approximate total time 8 h.

The entire FreezeTB workflow is intended to be completed in 1 day (Fig. 1). When there was a discrepancy between tNGS and pDST resistance determination, samples were re-extracted and run through the entire FreezeTB workflow a second time for validation. Discrepant results were also verified with sequencing data being run through the TB Profiler (v6.6.5) online pipeline (18). Whole genome sequencing was attempted with isolated DNA; however, it did not provide adequate coverage depth of all gene regions for comparison. The FreezeTB bioinformatics package is publicly available on **GitHub** at: https://github.com/jeremyButtler/freezeTB.

### Other bioinformatics analysis

Whole-genome sequences (WGS) were available for 79 of the 100 isolates from AKPHL via coordination with the Centers for Disease Control (CDC). The WGS of the 79 available isolates was run through the FreezeTB software as well as Mykrobe v0.10.0 (19, 20) and compared with FreezeTB using our targeted primer set. Discrepant results had WGS additionally run through PhyResSE v1.0 for further validation (21). Spoligotypes generated were uploaded into MIRU-VNTR*plus* (http://www.miru-vntrplus.org/) (22, 23) to generate a phylogenetic tree (with a maximum locus difference within a clonal complex of DLV 2).

### Statistical analysis

Percent agreement was calculated using Microsoft Excel (v16.98). Observer agreement with kappa was generated using the “Quantify agreement with kappa results” online tool (Graphpad Software, Boston, MA, USA; https://www.graphpad.com/quickcalcs/kappa2/) and interpreted based on previous methods (24).

### Cost approximation

Price per sample was based on laboratory supply costs listed on manufacturers’ websites from purchasing within the United States. The specific reagents and flow cells used for sequencing were included, as these are the main drivers of cost. Cost per sample did not include consumables such as pipette tips, gloves, PCR strips, or shipping and handling. The cost of DNA extraction was not included, as this can vary based on the sample type.

## RESULTS

DNA extraction and subsequent sequencing data were successfully generated on 98% (*n* = 98/100) of sputum culture isolates using the FreezeTB laboratory workflow and software. The two isolates that were not successful had low DNA; DNA was re-extracted from the remaining culture; however, the sequencing quality was poor. The other 98 isolates had complete coverage of gene targets with at least 50× depth of coverage.

Mutations associated with drug resistance are shown in Table 2. The most common mutation identified was katG_p.Ser315Thr, which is associated with INH resistance.

**Fig 2:**
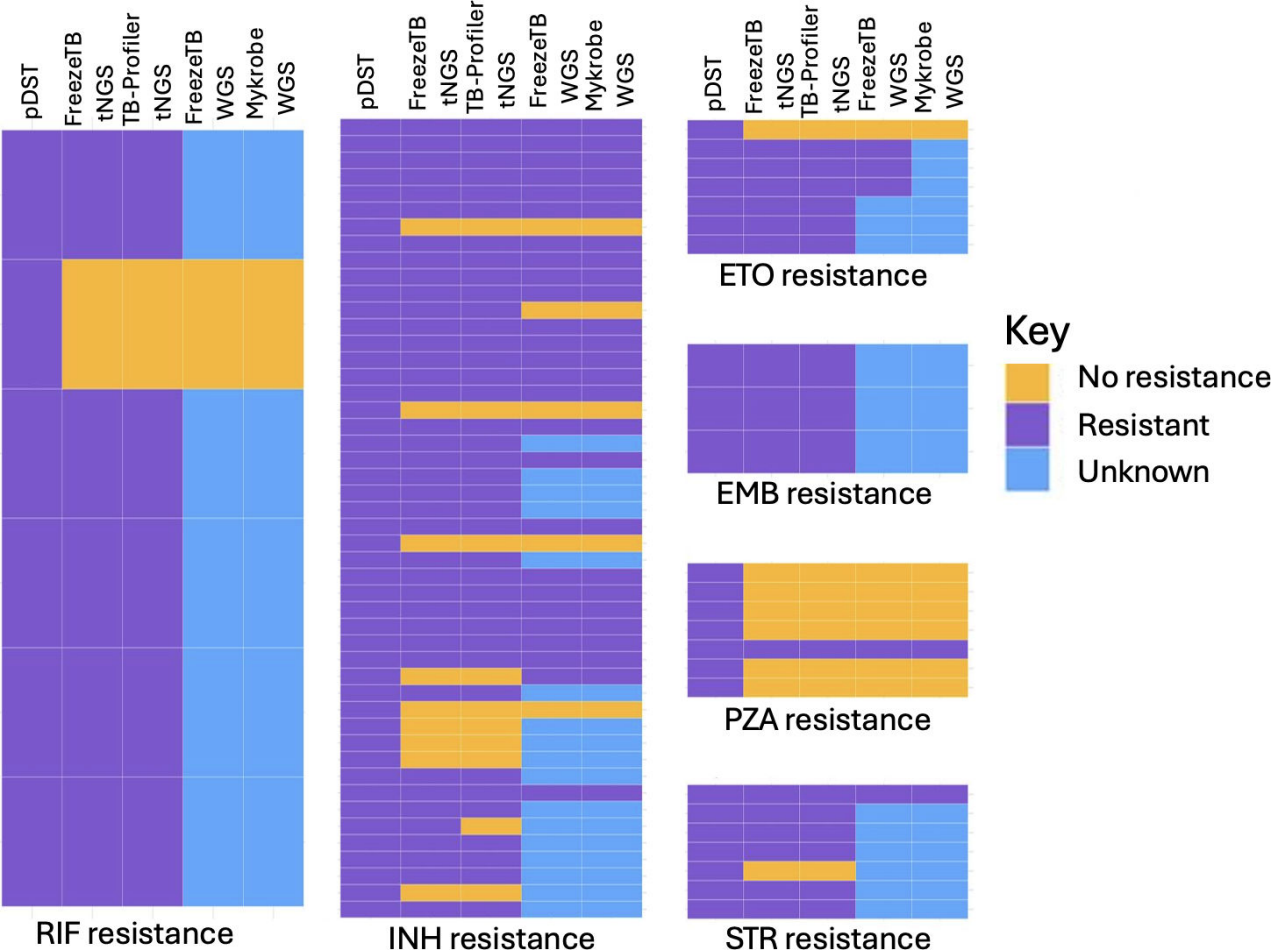
The results of pDST, tNGS (*n* = 98), and WGS (*n* = 78) of Alaskan pulmonary TB isolates that were run through the FreezeTB laboratory and software workflow successfully. Abbreviations: EMB: Ethambutol; ETO: Ethionamide; INH: Isoniazid; PZA: Pyrazinamide; STR: Streptomycin; tNGS: Targeted Next Generation Sequencing; WGS: Whole Genome Sequencing.

Compared with identified resistance on pDST, FreezeTB workflow identified 83% of rifampin (*n* = 5/6), 79% of isoniazid (*n* = 38/48), 86% of streptomycin (*n* = 6/7), 100% of ethambutol (*n* = 3/3), and 86% of ethionamide resistance (*n* = 6/7), but only 14% of pyrazinamide resistance (*n* = 1/7) (Fig. 2). Using Cohen’s kappa for agreement, FreezeTB had almost perfect agreement for RIF (0.90) and EMB (1.00), substantial agreement for ETO (0.78) and INH (0.80), moderate agreement for STR (0.56), and slight agreement for PZA (0.20).

FreezeTB tNGS was 98.7% (*n* = 77/79) in agreement per sample with WGS run through the FreezeTB software platform and 96.2% (*n* = 76/79) in agreement per sample with WGS run through Mykrobe (20). FreezeTB tNGS missed one case of INH resistance (katG_p.Ser315Thr) identified by WGS on both software programs, missed one case of RIF resistance (borderline resistance rpoB_p.Leu430Pro with no RIF resistance on pDST) identified on both software programs, and caught one case of STR resistance (rpsL_p.Lys88Gln) that was identified on WGS through the FreezeTB software but not Mykrobe- and had no pDST resistance on testing. These results were verified using PhyResSE, which agreed with WGS results from Mykrobe.

**Fig 3:**
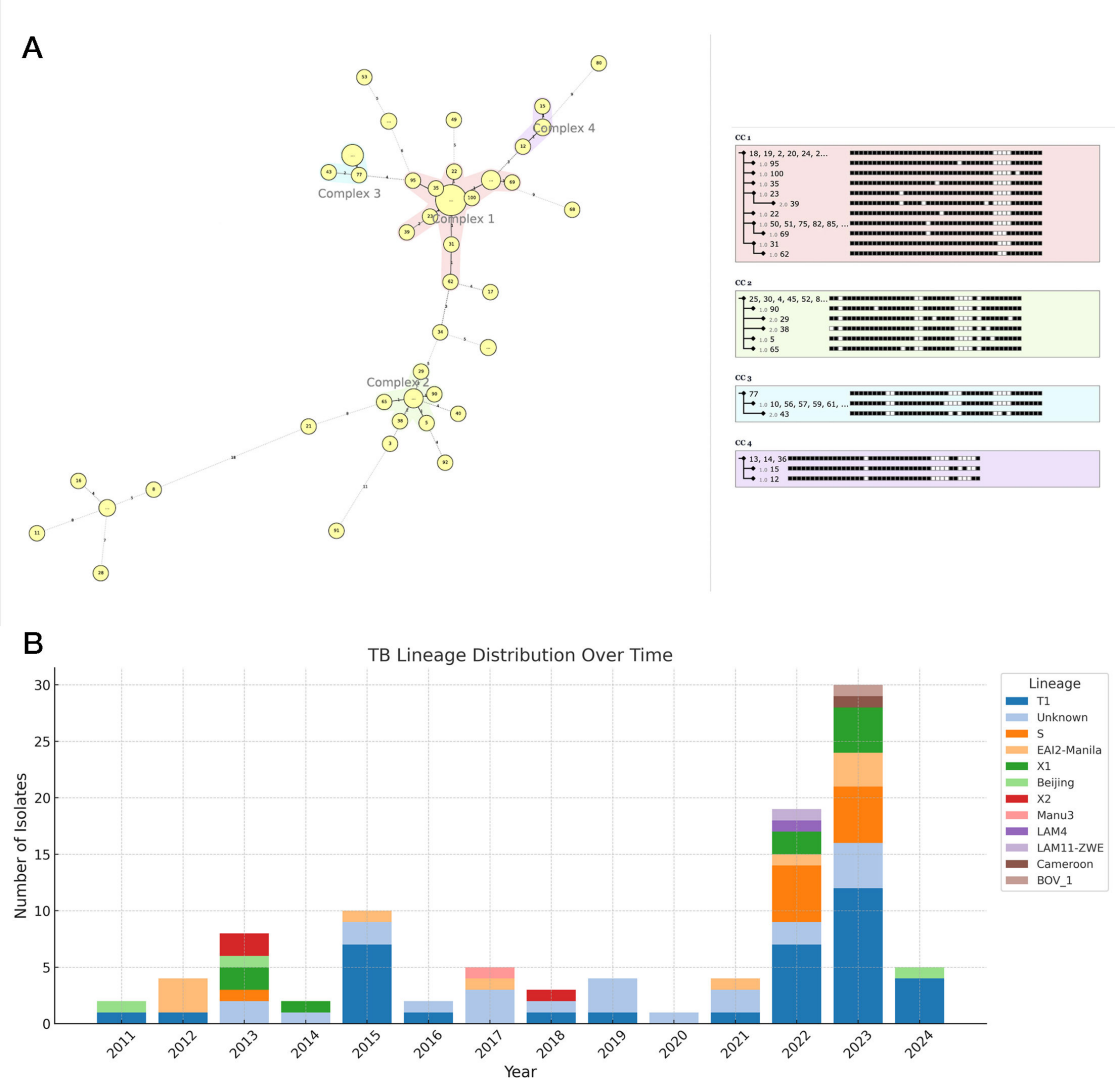
(**A**) Minimum spanning tree depicting clonal complexes of Alaskan Pulmonary TB isolates as determined by FreezeTB spoligotyping. (**B**) Distribution of spoligotypes selected and sequenced from 2011 to 2024 in Alaska.

Specific lineage identification was provided for 78 of the isolates by spoligotyping that is included as an option in FreezeTB and the primer set (Table 1); Lineage 4 (L4: Euro-American) was the dominant lineage, with 37 L4 T1 sublineage, but there were a diversity of other lineages including EIA2-Manila, S, T2, and Beijing, with distribution patterns changing of isolation year (Fig. 3B; Table S1). The remaining 22 isolates generated a spoligotype that was “Unknown” or “None.” Sputum culture from one TB case was identified as *Mycobacterium bovis* by FreezeTB analysis and confirmed with TB-Profiler, Mykrobe, and consistent with ASPHL documentation. Figure 3A displays all lineages as a phylogenetic tree based on spoligotype clustering. Spoligotypes identified four clonal complexes in our data set that spanned the sampling period (2011–2024) but clustered only weakly by year of isolation (Fig. 3A and B). The cost per sample for tNGS was $29.09 when considering the purchase of flow cell, barcoding kit, flow cell wash kit, primer pool, and master mix (including polymerase). To ensure quality results, only 40 samples are run on a flow cell (20 samples per run with one wash in between). Prices are accurate as of July 15, 2025 (Table 3).

## DISCUSSION

We designed and validated FreezeTB, an open-source, lab-designed tNGS assay using a portable nanopore sequencing platform for the determination of TB drug resistance and *M. tuberculosis* lineage. In this selective, retrospective analysis of TB cases (*N* = 100) in Alaska, we were able to identify drug-resistant mutations consistent with WGS. This laboratory workflow and OS software is designed to have minimal initial investment in sequencing equipment and be low-cost per sample, at approximately $30 at the time of publication. In addition, the workflow can be completed within one working day (under 8 h) after sample growth (culture) or collection. This work builds upon previous studies of commercial options (11, 25) with the goal of making rapid, low-cost *M. tuberculosis* drug resistance sequencing tools available worldwide on multiple specimen types (5). A recent meta-analysis of nanopore sequencing including five studies for drug resistance reported an overall sensitivity of 91% for INH resistance, 93% for RIF resistance, 84% for EMB resistance, 80% for STR resistance, and 78% PZA resistance, which is comparable with our results of 79% for INH, 83% for RIF, 100% for EMB, and 86% for STR (26). The percent agreement with phenotypic resistance to PZA was lacking at only 17%, and although tNGS of *M. tuberculosis* for PZA resistance has often had a lower sensitivity in other studies (27), our study is still substantially lower than expected. Interestingly, WGS identification of PZA resistance was consistent with our FreezeTB findings, which may indicate that pDST was over-calling resistance. False positive results of PZA resistance on pDST are a known phenomenon due to the acidic environment required by PZA, which can be impacted by inoculum size. Studies with higher sensitivity for PZA often utilize WGS as a reference standard to help account for this phenomenon. The WHO has evaluated and endorsed three commercial kits for tNGS of *M. tuberculosis,* but with different drugs meeting class-based performance criteria. The AmPORE TB kit from ONT met the criteria for RIF, INH, fluoroquinolones (FQ), linezolid (LZD), amikacin (AMI), and STM; the Deeplex-MYC TB kit for RIF, INH, PZA, EMB, FQ, bedaquiline, LZD, clofazimine, AMI, and STM, and TBSeq (ShengTing Biotech) for EMB (5, 28).

For the detection of INH, RIF, ETH, STM, and ETH, FreezeTB offers a diagnostic platform that is superior to pDST when considering time to test result, cost, and agreement with WGS. A recent study demonstrated the median time to sputum culture pDST result to be 35 days (29), highlighting the benefit of the rapid workflow utilized in FreezeTB of under 1 day from sample collection to resistance report. In addition, the price of sequencing supplies per sample, combined with the ability to perform on a low-cost, portable sequencing machine, makes it a rapid and affordable option. This study provides a robust resource to low-income countries that may face profound difficulties in controlling endemic TB, given that the current commercial options, while self-contained and proven, are many times more expensive. Providing a workflow with discrete components ordered through widely used commercial sources supports adaptability through every step of the presented protocol. Although pDST was previously the gold standard for the identification of drug resistance, it is time-consuming, costly, provides limited drug information, and can have variability in accurate resistance identification based on methodology.

There are several major limitations to the scope of this work. First, the 100 isolates analyzed were samples biobanked over the last 15 years that were specifically selected to bias toward having DR/MDR mutations present to test the detection power of FreezeTB. Hence, the results do not represent a comprehensive epidemiologic picture of Alaskan TB. Second, WGS was only available on a subset of samples for comparison. FreezeTB performance was not compared with the other commercial tests available, such as AmPORE TB or Deeplex Myc-TB, which would be a worthwhile study in the future. Finally, we provided a cost approximation rather than a formal cost-effectiveness analysis, as this was outside the scope of this project, making it inappropriate to directly compare our cost approximation to all other estimates, which may be inclusive of consumables, equipment, staffing, and overhead.

In conclusion, FreezeTB is a rapid and accurate method able to identify RIF, INH, ETH, EMB, and STM mutations associated with drug resistance and is designed to detect all drug mutations defined by the WHO as drug resistant. FreezeTB’s ability to offer affordable tNGS of *M. tuberculosis* without the need for internet makes it a valuable option for many TB high-burden, resource-constrained settings needing retrospective epidemiological analyses to inform local TB control strategies. Furthermore, as FreezeTB can quickly and easily be adapted to identify new mutation targets by incorporating additional primer pairs and updating the software’s tsv reference file for mutations, it provides a data-driven foundation for developing new tNGS sequence-based diagnostics for DR-TB.

## Data Availability

FreezeTB sequence data associated with this study is available in NCBI SRA under BioProject PRJNA1338723.

## SUPPLEMENTAL MATERIAL

Supplemental material spectrum.02335-25-s0001.docx Fig. S1 and S2; Table S1.

ASM does not own the copyrights to Supplemental Material that may be linked to, or accessed through, an article. The authors have granted ASM a non-exclusive, world-wide license to publish the Supplemental Material files. Please contact the corresponding author directly for reuse.
