# Supplementary material for: Application of FreezeTB, a targeted nanopore sequencing assay, for identification of drug resistance and lineages among pulmonary tuberculosis cases in Alaska
Source: Microbiol Spectr. 2025 Nov 17;14(1):e02335-25. doi: 10.1128/spectrum.02335-25 (PMC12772309; doi:10.1128/spectrum.02335-25)
Supplement: Supplemental material — Fig. S1 and S2; Table S1. [file spectrum.02335-25-s0001.docx]

**Supplemental Material 1. Sputum Specimens Decontamination and DNA Extraction**

***Decontamination***

**Preparation of working solutions before beginning:**

**NALC-NaOH-Sodium citrate solution:** Combine 0.25 grams of NALC powder and dissolving fully in a 50 mL solution of 2% NaOH and 1.45% sodium citrate (a 1 liter stock solution of NaOH-sodium citrate can be made by combining 20 grams NaOH and 14.5 grams sodium citrate dihydrate and adding 1 Liter of distilled water). Ensure the solution is completely dissolved.

**Phosphate buffer** (0.067 M): Add 4.74 grams of disodium phosphate and 4.54 grams of monopotassium phosphate and add 1 Liter of distilled water. Ensure the solution is completely dissolved.

**Method:**

1. Take 700 microliters of direct sputum or sputum culture liquid isolate and add 700 microliters of NALC-NaOH0-sodium citrate solution in 1.5 or 2.0 mL microcentrifuge tubes. Process each sample individually and be careful not to cross-contaminate.
2. Vortex the tubes for and incubate at room temperature for ten minutes.
3. Centrifuge the tubes at 15,000 g for three minutes. Discard the supernatant by carefully pipetting off the supernatant without disturbing the pellet.
4. Add 1 mL of phosphate buffer (0.067 M) to resuspend the pellet.
5. Centrifuge at 15,000 g for three minutes. Carefully discard the supernatant again without disturbing the pellet.
6. Proceed to manual extraction (if not done prior to receipt of samples, heat inactivation at 80-85C, 30’, or higher/longer, according to lab-specific biosafety procedures; *Note longer and higher heat kill temperatures may result in DNA degradation*).

***DNA Extraction***

**Preparation of working solutions before beginning:**

**PBS+ 0.1% Tween:** Add 1 mL Tween-80 to 9 mL phosphate-buffered-saline (PBS) and vortex. Add 0.5 mL 10% Tween-80 to 50 mL PBS and vortex.

**Method:**

1. ​​After decontamination (above), resuspend each (decontaminated) pellet in 200 µL PBS + 0.1 % Tween-80 + 20 µL Proteinase K and incubate at 56 °C for 10 minutes, including extraction PC and extraction NTC.
2. ​​(Optional step if not done prior)​ Heat kill step**:** incubate the tubes at 95 °C for 15 minutes.
3. ​​Invert mix Reagent DX before use. Add 1 µL, mix by pipetting and transfer the solution to the Qiagen Pathogen Lysis Tubes L bead tube.
4. ​​Vortex the tubes for 1 minute.
5. ​​To each tube, add 250 µL of Buffer ATL and 20 µL of Proteinase K and mix shortly by vortexing for 2 seconds.
6. ​​Incubate at 56 °C for 10 minutes.
7. ​​While the samples are being incubated, set up 350 µL of Buffer AL and 350 µL of 100% ethanol in a fresh 1.5 mL Eppendorf tube per sample.
8. ​​After incubation (step 6) vortex the sample and transfer 350 µL from the bead tube (avoiding transfer of beads) to the tube containing Buffer AL and 100% ethanol (step 7). Vortex for 15 seconds.
9. ​​Transfer 600 µL to a DNeasy spin column and centrifuge at 6,000 xg for 1 minute. Discard the flowthrough from the collection tube (by pouring) and return the insert into the same collection tube.
10. Transfer the remainder of the sample to the DNeasy spin column and centrifuge at 6,000 xg for 1 minute. Transfer the column to a new collection tube.
11. Add 500 µL Buffer AW1 to the column and centrifuge at 10,000 xg for 1 minute. Transfer the column to a new collection tube.
12. Add 500 µL Buffer AW2 to the column and centrifuge at 20,000 xg for 3 minutes.
13. Transfer the column to a fresh 1.5 mL Eppendorf tube and add 20 to 50 µL of Buffer AE to the column membrane. Incubate at room temperature for 1 minute.
14. Centrifuge the sample at 10,000 xg for 1 minute. Discard the column and keep the eluate in the Eppendorf tube.

The eluate is ready to be used for the multiplex PCR or can be stored at 4°C for one week, or at -20°C for longer.

### **Supplementary Material 2. How FreezeTB Works**


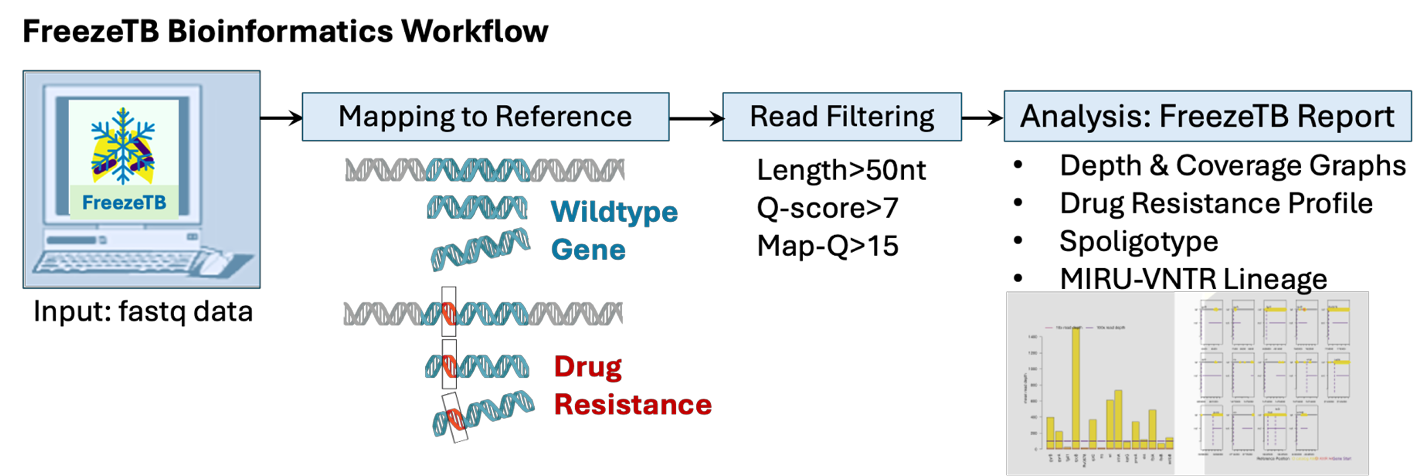


**Supplemental Figure 1:** Overview of FreezeTB bioinformatic workflow. Raw data (pod5) is demultiplexed and basecalled by ONT MinKNOW using a super high-accuracy model, and fastq files are input for FreezeTB software. FreezeTB maps sequence reads to a reference genome (NC000962.3; *M. tuberculosis* strain H37Rv), filters low quality mapped reads, and generates a FreezeTB report of read depth and coverage for each target gene, the strain’s drug resistance profile and lineage (octal that can be converted to spoligotype; and MIRU-VNTR lineage).

***Abbreviations:*** *nt: nucleotide; Q-score: Quality score.*

FreezeTB is a program that detects AMRs, MIRU-VNTR lineages, spoligotypes, and builds a consensus from *M. tuberculosis* reads. It includes a GUI which is currently written using tcltk^30^ that uses minimap2^31,32^ to map reads to the NC000962.3 reference genome. The GUI will display drug resistance detected, the spoligotype, the mean read depth graph (made by R), and the coverage graph (made by R).

*Read Mapping*

In the FreezeTB GUI reads are mapped using minimap2 or an internal read mapper. For the command line program, you can provide a sam file or use the internal read mapper. The internal read mapper converts the reference genome and a reads sequence to 13mers. We sort the references 13mers and then map the reads 13mers to every reference 13mer that matches. Neighboring 13mers in both the query and reference are then merged into chains. Neighboring chains having a gap under 25% of the query length and that increase the total chain score (bases in chain + [gap size * -1]) are merged. For the chain merging step, chains are merged from the last query and reference positions to the first query and reference position. Gaps between the chains are then filled with a Needleman pairwise aligner using a gap opening score of -10, a gap extension score of -1, and an EDNA full scoring matrix. The best local alignment is then found by selecting the highest scoring sub alignment (same scoring as Needleman) in the complete alignment. These steps are repeated until all possible merged chains, which have at least 20% of the query bases have been aligned. Only the best scoring alignment for the forward and reverse complement read sequence is kept. The read is considered unmapped if the best alignment has a score under 40% of the maximum local (bases aligned) score.

*Read depth and coverage reports*

To get the read depths we first remove reads with a mapping quality under 15 (minimap2 only), a mean Q-score under 7, a median Q-score under 7, and reads that are less then 50 bases long (Figure Sup. 1). The remaining reads are then used to build a read depth histogram, which is then used to find the mean read dept for each gene. The mean read depth is then used to build the mean read depth and coverage graph (with at least 10x read depth) is using R.^33^

#### Primer masking

FreezeTB includes an optional primer masking step. In this step primers are masked using the position each primer maps on the H37Rv reference genome (NC000962.3).

#### Consensus building

For consensus building Figure Sup. 1, we use an majority consensus step that is somewhat similar to Ivar.^34^ We count the number of SNPs, matches, and insertions for each position that had a quality score of at least seven. Next, we collapse the consensus by keeping the most supported SNP, match, deletion or insertion. We then mask SNPs and matches supported by a low percentage of reads (< 50%; support / (number matches + number SNPs)). We also remove insertions and mask deletions in the consensus that are supported by a low percentage (< 70%; (support / mapped reads) of mapped reads. For insertions, the number of mapped reads is from the neighbor with the most reads. Finally, we fragment the consensus by removing any position that has less than 10x read depth. We then remove any fragment that is under 50 bases long.

#### AMR detection

For AMRs detection (Figure Sup. 1) we use a tsv version of the WHOs 2023 TB mutation catalog^15^ that has all grade three, four, and five AMRs removed. We find potential AMR variants for each sequence with the sequence’s reference mapping coordinates. For AMR variants with amino acid sequence, we convert the sequence’s AMR region into amino acids. We then check if the sequence's nucleotide or amino acid sequence matches the AMR variant. Next, we detect and remove false positive AMRs by discarding variants where the sequence's AMR region length differs from the AMR variants length.

We also check for frame shifts and loss of function AMRs (disable this for R9.4 flow cells). First, we remove any small indels (under five bases) in homopolymers larger then 3. Then we search for indels in the AMR region, count the number of indels and check if the number of deletions minus the number of insertions are divisible by three ((deletions – insertions) % 3 = 0). If the insertions and deletions are not a multiple of three, then the read or position is treated as having a loss of function. For loss of function entries, we also look for early stop codons, lost stop codons (when possible), and lost start codons (when possible).

#### MIRU-VNTR lineages

To detect MIRU-VNTR lineages (Figure Sup. 1) we identify primer sites on the reference (NC000962.3) using the sequence’s mapping coordinates. We find the lineage for the site by comparing the sequence’s repeat region to a modified MIRU-VNTR table from MIRUReader.^35^ To account for indel errors we allow a 15-base difference between the sequence’s length and the actual lineages length.

#### Spoligotyping

To detect spoligotypes we find the direct repeat region from NC000962.3 in the consensus using its mapping coordinates. We then build the spoligotype barcode by mapping each internal spacer sequence to the direct repeat region in the sequence using our kmer scan. Next, we search the lineage database (csv) from TBProfiler for lineages having the same spacers.

The kmer scan works by dividing the reference genome into 50 base long windows that share a 25-base overlap with their neighboring windows. If the spacer and window share at least 50% of the kmers, we use a Waterman Smith alignment, with a EDNA full matrix to align the spacer to the window. To count a spacer as mapped we require a score 90% of the maximum expected score.

**Supplementary Table 1: Lineage determination of Alaskan Pulmonary TB Isolates via Spoligotyping.** Lineage classification by spoligotyping (Dra/b locus sequencing, N=98 isolates, including Unknown determination).


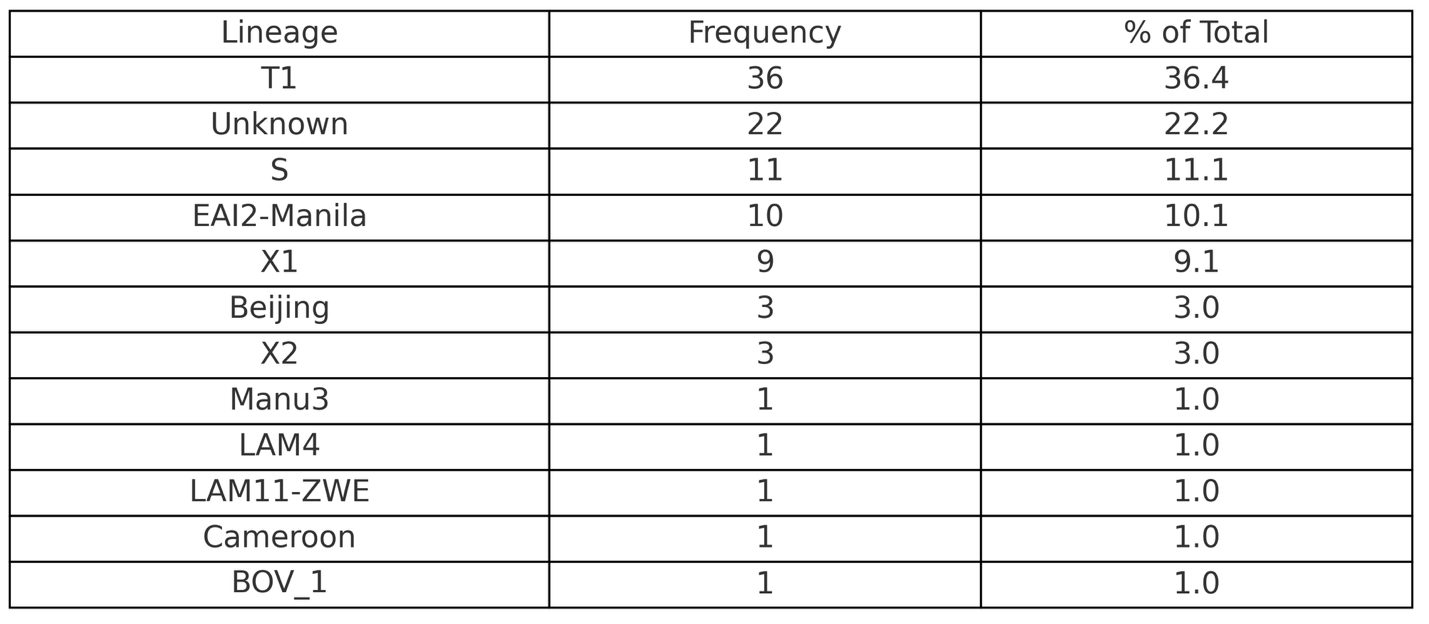


**Supplementary Figure 2.** Phylogenetic Tree of Alaskan Pulmonary TB Isolates via Spoligotyping.


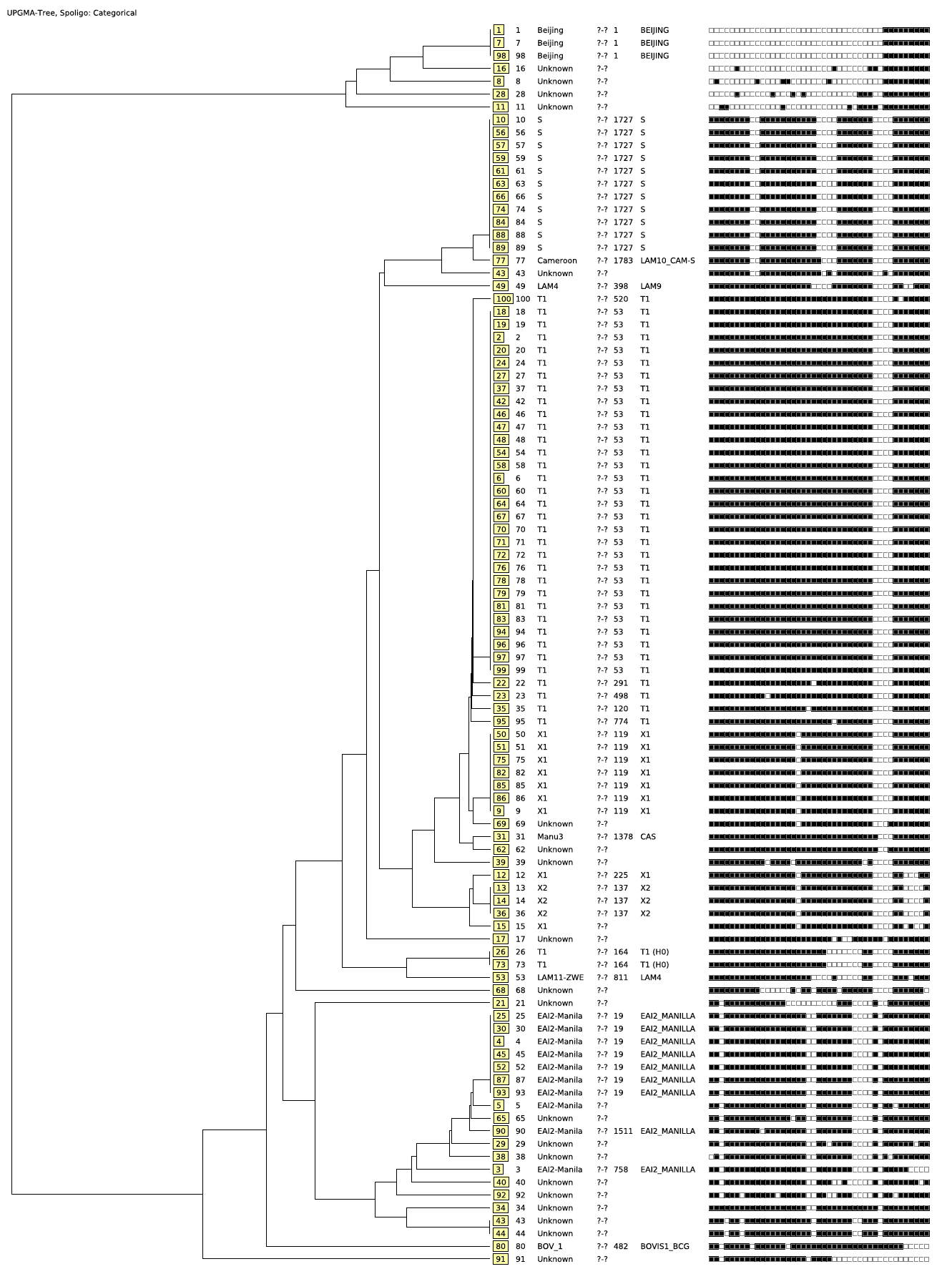


**REFERENCES**

30. Grosjean P. 2022. R: a GUI API for R. UMONS, MONS. Available from: <http://www.sciviews.org/SciViews-R>

31. Li H. 2018. Minimap2: pairwise alignment for nucleotide sequences. Bioinformatics 34:3094–3100. https://doi.org/10.1093/bioinformatics/bty191

32. Li H. 2021. New strategies to improve minimap2 alignment accuracy. Bioinformatics 37:4572–4574. https://doi.org/10.1093/bioinformatics/btab705

33. Team RC. 2020. R: a language and environment for statistical computing. R Foundation for Statistical Computing, Vienna, Austria. https://www.R-project.org.

34. Grubaugh ND, Gangavarapu K, Quick J, Matteson NL, De Jesus JG, Main BJ, Tan AL, Paul LM, Brackney DE, Grewal S, Gurfield N, Van Rompay KKA, Isern S, Michael SF, Coffey LL, Loman NJ, Andersen KG. 2019. An amplicon-based sequencing framework for accurately measuring intrahost virus diversity using PrimalSeq and iVar. Genome Biol 20:8. https://doi.org/10.1186/s13059-018-1618-7

35. Tang CY, Ong R-H. 2020. MIRUReader: MIRU-VNTR typing directly from long sequencing reads. Bioinformatics 36:1625–1626. https://doi.org/10.1093/bioinformatics/btz771
